# Absence of Collagen Flowers on Electron Microscopy and Identification of (Likely) Pathogenic *COL5A1* Variants in Two Patients

**DOI:** 10.3390/genes10100762

**Published:** 2019-09-27

**Authors:** Chloe Angwin, Angela F. Brady, Marina Colombi, David J. P. Ferguson, Rebecca Pollitt, F. Michael Pope, Marco Ritelli, Sofie Symoens, Neeti Ghali, Fleur S. van Dijk

**Affiliations:** 1 National Complex Ehlers-Danlos Syndrome Service London, North West Health Care NHS Trust, Harrow HA1 3UJ, UK; chloeangwin@nhs.net (C.A.); angela.brady@nhs.net (A.F.B.); neeti.ghali@nhs.net (N.G.); 2 Division of Biology and Genetics, Department of Molecular and Translational Medicine, University of Brescia, 25123 Brescia, Italy; marina.colombi@unibs.it (M.C.); marco.ritelli@unibs.it (M.R.); 3 Nuffield Department of Clinical Laboratory Sciences, University of Oxford, John Radcliffe Hospital, Oxford OX3 9DU, UK; david.ferguson@ndcls.ox.ac.uk; 4 Department Biological & Medical Sciences, Oxford Brookes University, Oxford OX3 0BP, UK; 5 Connective Tissue Disorders Service, Sheffield Diagnostic Genetics Service, Sheffield S10 2TQ, UK; rebeccapollitt@nhs.net; 6 Department of Dermatology, Chelsea and Westminster Hospital, London, SW10 9NH UK; fmandhpope@googlemail.com; 7 Center for Medical Genetics, Ghent University Hospital, B-9000 Ghent, Belgium; Sofie.Symoens@UGent.be

**Keywords:** classical Ehlers-Danlos Syndrome, electron microscopy, collagen flowers, *COL5A1*

## Abstract

Two probands are reported with pathogenic and likely pathogenic *COL5A1* variants (frameshift and splice site) in whom no collagen flowers have been identified with transmission electron microscopy (TEM). One proband fulfils the clinical criteria for classical Ehlers-Danlos syndrome (cEDS) while the other does not and presents with a vascular complication. This case report highlights the significant intrafamilial variability within the cEDS phenotype and demonstrates that patients with pathogenic *COL5A1* variants can have an absence of collagen flowers on TEM skin biopsy analysis. This has not been previously reported in the literature and is important when evaluating the significance of a TEM result in patients with clinically suspected cEDS and underscores the relevance of molecular analysis.

## 1. Introduction

The Ehlers-Danlos syndromes (EDS) consist of 13 subtypes with overlapping features including joint hypermobility, skin, and vascular fragility and generalised connective tissue friability [1,2]. Current major criteria for classical EDS (cEDS) are (1) skin hyperextensibility and atrophic scars and (2) joint hypermobility. Minor criteria are easy bruising, soft doughy skin, skin fragility, molluscoid pseudotumors, subcutaneous spheroids, hernia(s), epicanthal folds, complications of joint hypermobility, and an affected first degree relative. The minimal criteria for a diagnosis of cEDS are major criterion 1 plus either major criterion 2 or 3 of the 9 minor criteria [3].

In patients who satisfy the main criteria of cEDS according to the Villefranche criteria [4], the variant detection rate in either *COL5A1* or *COL5A2* is over 90% [5,6]. However, intrafamilial variability in classical EDS has been reported [7]. There are 194 reported unique variants reported in the *COL5A1* gene [8]. These genes encode collagen type V, a fibrillar heterotrimer ([α1(V)]_2_ α2(V)) that is present in a wide variety of tissues but is particularly prevalent in bone, skin and tendon [9]. Collagen type V accounts for approximately 5% of total body collagen and has a role in maintaining the deposition and structure of other more abundant collagens, particularly collagen type I [9]. Rarely, a diagnosis of cEDS is due to dominant variants in *COL1A1* or *COL1A2* [3,10,11]. 

For many years, skin biopsies for electron microscopy (EM) have been recommended as a first line of investigation to confirm or exclude a diagnosis of cEDS [3,12]. This was due to the occurrence of collagen flowers visible by EM. Collagen flowers in individuals with classical EDS were described by Vogel et al. who reported collagen fibrils with an abnormally large diameter and a highly irregular and lobulated contour interspersed with normal appearing fibrils with a mean diameter larger than that of collagen fibrils in normal skin [13]. A longitudinal section showed that the large atypical fibrils were seen to be poorly integrated filamentous aggregates. Variation in frequency of very large highly irregular fibrils differed per patient but in general constituted approximately 5% [13]. These very large highly-irregular fibrils are often described as longitudinally splayed and loosely packed fibrils, which in cross section produce the collagen flower pattern. Although it is known that collagen flowers can be found in other collagen disorders including osteogenesis imperfecta and Ullrich congenital muscular dystrophy, typical collagen flowers were thought to be invariably present in people with cEDS [3,14,15]. Given the high detection rates of pathogenic variants in cEDS, current recommendations are that electron microscopy based on a skin biopsy should no longer the first line of investigation but could be used to clarify inconclusive molecular results, or to guide further testing if initial molecular testing is negative [3]. Interestingly, it was mentioned that “the absence of typical collagen flowers would go against the diagnosis, as there are no known reports of patients with type V collagen abnormalities without collagen flowers on EM” [3]. Here, we report for the first time two patients, one fulfilling the clinical diagnosis of classical EDS and one not, with (likely) pathogenic variants in *COL5A1* and absence of collagen flowers on EM.

## 2. Materials and Methods

### 2.1. Skin Biopsy

Skin biopsies in probands 1 and 2 were taken in the form of a punch biopsy from the medial surface of the arm. P1 was 44 years and P2 was 39 years when the skin biopsy was performed. The sample for transmission electron microscopy (TEM) (3 mm) was preserved in 4% glutaraldehyde in 0.1 M phosphate buffer. This publication does not constitute research and does not require formal Research Ethics approval or Research and Development Approval as stipulated by the UK Policy Framework for Health and Social Care Research and the Health Research authority decision tool.

### 2.2. DNA Analysis 

Sanger sequencing of the *COL5A1* and *COL5A2* genes and MLPA of the *COL5A1* gene was performed in proband 1. Sanger sequencing and MLPA of the *COL3A1* gene was performed in proband 2. Proband 2 was included in a large research project aimed at identifying new pathogenic variants with next-generation sequencing in patients with an EDS phenotype [16]. The sequencing data of patient 2 have been deposited to the EDS and OI databases: https://www.le.ac.uk/genetics/collagen.

## 3. Clinical Report

### 3.1. Family 1

**Figure 1:**
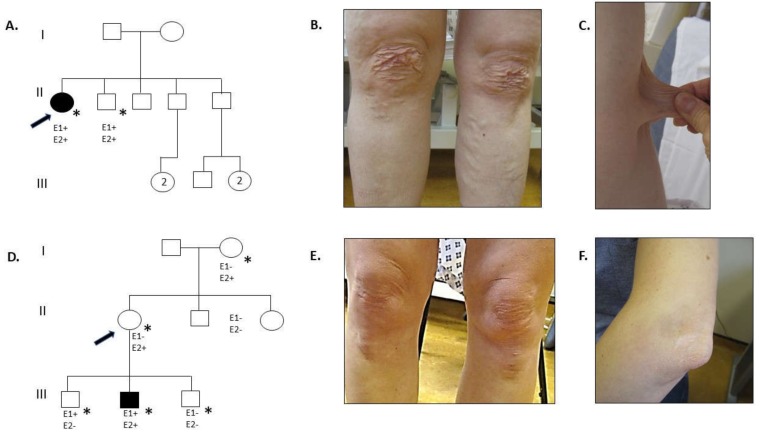
Pedigrees and clinical photographs of probands 1 and 2. (**A**) Family pedigree of proband 1. E1 denotes clinical diagnosis of cEDS, E2 denotes presence of (likely) pathogenic *COL5A1* variant, * denotes documented evaluation. Shading denotes having a clinical diagnosis of cEDS and presence of (likely) pathogenic *COL5A1* variant. (**B**) Knee area of proband 1 demonstrating redundancy of skin, atrophic and widened scarring and varicose veins. (**C**) Hyperextensible skin at the elbow in proband 1. (**D**) Family pedigree of proband 2. (**E**) Knees area of proband 2 show no atrophic scarring or haemosiderin deposition but minor redundancy of skin. (**F**) Widened, atrophic scar at the elbow.

Proband 1 is a 47-year-old woman, born at 36 weeks gestation, with a childhood history of clumsiness, tissue fragility, and joint hypermobility (Figure 1A–C). She would bruise easily and recalls some episodes of significant swelling after mild injury, occasionally requiring drainage when this involved the knees. There was no history of dislocation or fracture. As an adult she reports myalgia and arthralgia in the ankles, hips, wrists, and shoulders. Other medical history includes endometrial polyps removed without complication at age 32, varicose veins treated surgically at age 41, hiatus hernia, diverticular disease, a urethral cyst, and hypotension associated with fainting episodes. Neurocardiology investigations found a high tendency towards POTS (postural orthostatic tachycardia syndrome) with no evidence of autonomic failure. Cardiac investigations have shown right bundle branch block and trivial aortic regurgitation, tricuspid regurgitation, and mitral regurgitation.

She fulfilled the criteria for cEDS on physical examination. There were no craniofacial dysmorphic features except for epicanthic folds. She had soft, doughy, and hyperextensible skin—particularly at elbows, neck, and knees—with redundancy at the knees and Achilles tendons. She had scarring over the forehead, wrist, and lower legs. Lower leg scars were widened and atrophic with erythema and hemosiderin deposition. There were thread veins and varicose veins in the lower limbs. Her feet had bilateral hallux valgus deformities with piezogenic papules. Hands had bilateral hitchhikers’ thumb and increased palmar markings. She had generalized joint hypermobility (gJHM) with a (Beighton score 5/9). 

Family history: The proband’s parents, four older brothers and their children are healthy with no reported features of cEDS. One brother was tested for the *COL5A1* variant as well. He did not meet the criteria for cEDS and was found not to have the *COL5A1* variant. 

### 3.2. Family 2

**Figure 2:**
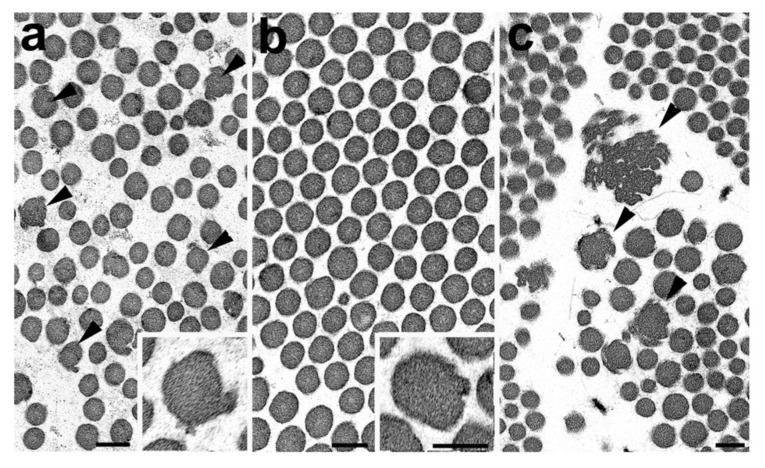
Electron micrographs of the reticular dermis of skin biopsy from proband 1 (**a**), proband 2 (**b**) and a case of classical EDS (**c**). Bars represent 100nm. (**a**) Cross section of collagen fibres from proband 1 showing a number of fibres presenting an irregular outline (arrowheads). Insert: detail of an irregular fibre. (**b**) Cross section through a clump if collagen fibres of proband 2 showing a relatively normal circular outline although very rare slightly irregular fibres were observed (insert). (**c**) Cross section of collagen fibres from a case of classical EDS showing a number of irregular fibres and large collagen flowers (arrowheads).

Proband 2 is a woman who was diagnosed with an acute, spontaneous, left carotid artery dissection presenting with left sided headache, and Horner’s syndrome at 37 years old (Figure 2D–F). This was successfully treated conservatively. She had no past medical history and was a non-smoker with normal cholesterol levels. Her past surgical history included bilateral bunion surgery at age 13 and three Caesarean sections, the first for fetal distress and the others as routine following without complication. 

On physical examination she did not have craniofacial dysmorphic features [17]. Her skin was mildly hyperextensible over face, neck, and elbows with delayed recoil and slight redundancy of the skin around the knees. The skin was not thin and no bruising was visible. A widened, atrophic scar at a site of trauma over the elbow and thin, well healed post-operative scars from bunion surgery were observed. There was bilateral hallux valgus She did not have gJHM. (Beighton score 3/9) and did not fulfil the current clinical criteria for cEDS. She had a normal echocardiogram.

Family history: The mother of the proband has the *COL5A1* variant and had a healthy childhood but developed hypertension later in life. Findings on clinical examination at the age of 81 were that of skin hyperextensibility with significantly delayed recoiling probably due to her advanced age, abdominal striae, a mild spinal scoliosis, and bilateral hallux valgus. The father of the proband had a history of joint hypermobility and hallux valgus and passed away at the age of 81. He had a healthy childhood and later in life developed hypertension, bowel cancer, skin cancer, and cardiovascular disease, requiring a double coronary artery bypass graft post myocardial infarction.

The proband has two siblings, a sister and a brother. There is no clinical information available regarding her brother. The proband’s sister has a cardiovascular history with bicuspid aortic valve requiring replacement, and aortic root dilatation. She does not have the *COL5A1* variant found in her mother and sister. 

The proband has three sons, all born by caesarean section. The oldest son, 9 years old, experienced foetal distress and oxygen deprivation in the womb, requiring emergency caesarean section and developed cerebral palsy with significant spasticity. On examination, there was minimal bruising, atrophic scarring over the forehead and occiput, mild hyperextensibility of the skin, and a Beighton score of 2/9 and therefore did not meet the current clinical criteria for cEDS. He had a normal echocardiogram and was found to have the *COL5A1* variant. The middle son, 7 years old, had had a healthy childhood. On examination he had hyperextensible skin, atrophic scarring on the forehead and lower legs, bruising over the shins and a Beighton score of 7/9 and therefore met the current clinical criteria for cEDS. He had a normal echocardiogram and he was found to have the *COL5A1* variant. The youngest son, 4 years old, had had a healthy childhood and did not have any clinical features of cEDS. He therefore did not meet the current clinical criteria for cEDS and was found not to have the *COL5A1* variant. 

## 4. Results

### 4.1. Skin Biopsy TEM Results

Proband 1: TEM was concluded to be relatively normal but showed some minor deformation of the outline of certain collagen fibrils (Figure 2a). No large collagen flowers were observed. Elastic fibres and fibroblasts appeared normal. 

Proband 2: TEM showed collagen clumps within the reticular dermis. Within these, the collagen fibrils themselves were of relatively even diameter and had a symmetrical circular outline (Figure 2b), although longitudinally sectioned fibres showed occasional kinking. No abnormal collagen flowers were seen within the biopsy (Figure 2b). The other structures including elastic fibres and fibroblasts were normal in appearance. The conclusion was one of a normal skin biopsy. After the molecular result, re-examination identified rare, slightly deformed fibrils (Figure 2b) with slight variation in collagen fibril diameter but no further abnormalities and no typical collagen flowers as described previously (Figure 2c). It should be noted that collagen flowers are concentrated within the reticular dermis and rarely observed in the papillary dermis. Therefore, care is required to ensure the correct area of the skin biopsy was examined.

### 4.2. DNA Analysis

Proband 1: DNA analysis identified the heterozygous *COL5A1* variant c.4414del, p.(Leu1472Serfs*16). This variant results in a pathogenic frameshift in exon 57 of *COL5A1* (NM_000093.4; NP_000084.3), predicted to result in a premature termination codon, nonsense-mediated RNA decay, and consequent type V collagen haploinsufficiency.

Proband 2: DNA analysis of *COL3A1* in which variants cause vascular EDS, did not identify pathogenic variants. The patient was referred for further sequencing as part of a large research project looking at Mendelian inheritance [16] and the likely pathogenic variant c.4068G>A; p.(Ala1356=) in the *COL5A1* gene was identified. This silent variant has been previously published is predicted to result in abnormal splicing of exon 51 of the *COL5A1* gene [7]. Splice site prediction software (Alamut visual software) indicated that the variant would destroy the splice donor site leading to disruption of normal splicing with skipping of exon 51, leading to the production of a shortened protein [7]. The same variant was identified in the mother of the proband as well as the proband’s two older sons, associating with the clinical diagnosis of cEDS. The variant was not identified in the youngest son (Figure 1B).

## 5. Discussion

We report two patients with a (likely) pathogenic *COL5A1* variant who did not have collagen flowers on electron microscopy although minor deformation of the outline of certain collagen fibrils were observed in proband 1, which are similar to the initial stage of collagen flower formation (cf Figure 2a,c). Large collagen flowers on electron microscopy have previously been used to confirm or exclude a clinical diagnosis of cEDS. One other report describes a 12-year-old boy with marked clinical features of cEDS, without collagen flowers on electron microscopy. Interestingly, investigation showed disorganisation, variation of fibril diameter, and irregular fibril outlines. However, this patient did not have molecular investigations, and the underlying molecular basis is unknown [18].

### 5.1. Phenotype

Proband 1 clearly fulfilled the criteria for cEDS. Proband 2 did not fulfil the clinical criteria for cEDS. Moreover, she presented with a vascular complication (carotid artery dissection) which is not a common feature in cEDS [19]. In a recent systemic review, 12/110 (11%) patients with *COL5A1/2* variants had vascular complications of which six had arterial dissection, three had arterial aneurysms, and one had intracerebral haemorrhage. It is not certain whether the vascular complication in this proband is linked to the *COL5A1* variant. It has been hypothesized that glycine substitutions near or at the C-terminal end of collagen type V, may predispose to vascular events [20], but at this point there is not enough evidence to support this hypothesis. 

There is clear intrafamilial variability across the generations of the family of proband 2, as the mother of the proband was 81 years old with skin hyperextensibility as the only major feature whereas one grandson with the same variant had hyperextensible skin and atrophic scarring, clearly fulfilling the clinical criteria for cEDS. Wide variability between family members with the identical *COL5A1* variant has been previously reported [6,17].

### 5.2. Transmission Electron Microscopy

In proband 1, who fulfilled the clinical criteria for cEDS, minor deformation of the outline of certain collagen fibrils were observed but no collagen flowers were present. The appearances were similar to early changes seen in fibrils in cEDS (Figure 2a,c). These changes were sufficient for the abnormality to be highlighted in the EM report but not significant enough to conclude that it concerned an abnormal biopsy. In proband 2, no collagen flowers or other abnormalities were observed. Unfortunately, the family members of proband 2, who had pathogenic *COL5A1* variant (mother and sons of proband), did not consent for a skin biopsy to be performed and as such no TEM results are available. While the presence or absence of collagen flowers is reported, it should also be noted that incidence and size/complexity of the collagen flowers can vary markedly between patients but the significance of this to the clinical features and the underlying genetic cause is unknown (Ferguson unpublished observations).

### 5.3. COL5A1 Variants

The variant identified in proband 1, who fulfils clinical criteria for cEDS, is a pathogenic frameshift variant in exon 57 predicted to result in a premature termination codon leading to haploinsufficiency. The likely pathogenic variant in *COL5A1* c.4068G>A identified in proband 2, who does not fulfil clinical criteria for cEDS, is a splice site variant and expected to result in skipping of exon 51 which is in-frame and as such would lead to a shortened protein and exert a dominant-negative effect. One variant was described by Symoens et al., *COL5A1* c.4068G>T which demonstrated skipping of exon 51 on mRNA analysis; p.(Gly1339_Ala1356del). The proband had skin hyperextensibility, atrophic scarring, joint hypermobility, and no history of vascular events. TEM was not performed [5]. Colombi et al, identified an identical variant. The proband in this case did not fulfil the clinical criteria for cEDS, and reported joint instability, gastrointestinal symptoms, and soft skin with a few small atrophic scars over the knees. She had historical generalised joint hypermobility. Predictive software (Alamut visual software) projected a similar functional outcome as the previous *COL5A1* c.4068G>T variant [7], both reduce the donor site strength to similar extents so a similar protein effect would be expected. According to the ACMG guidelines [21] this variant is classified as likely pathogenic fulfilling *PP3*: Multiple lines of computational evidence support a deleterious effect on the gene or gene product (conservation, evolutionary, splicing impact, etc.); *PM2*: Absent from controls (or at extremely low frequency if recessive) in Exome Sequencing Project, 1000 Genomes Project, or Exome Aggregation Consortium and *PS1*: Same amino acid change as a previously established pathogenic variant regardless of nucleotide change.

Hypotheses for this incomplete cEDS phenotype included protective lifestyle factors, potential for this variant to result in only partial activation of abnormal splicing of exon 51 and the existence of protective variants in other genes, which may counteract the loss of collagen V function during the deposition of structural collagens [7].

Of the 194 unique variants reported in the *COL5A1* gene, 31 have been reported as splice site variants [8]. Most pathogenic *COL5A1* variants (including splice site variants that introduce a premature stop codon) lead to haploinsufficiency for *COL5A1* mRNA. This is expected to be the case for the variant reported in P1. Structural variants exerting a dominant-negative effect are a minority and most commonly involve splice site variants resulting in exon skipping which is the case for the variant reported in P2 and variants that result in the substitution for glycine in the triple-helical region [22]. Collagen type V is thought to perform a regulatory function in collagen fibrillogenesis. It has been hypothesized that the final common pathway for all *COL5A1* variants is reduced availability of collagen type V, and that clinical phenotypes result from disrupted fibrillogenesis [5,8,23,24].

## 6. Conclusions

In conclusion, we present two probands with (likely) pathogenic *COL5A1* variants (frameshift and splice site) in whom no collagen flowers were identified, although minor deformation of the outline of certain collagen fibrils was observed in proband 1. Proband 1 fulfils the clinical criteria for cEDS but proband 2 does not and presents with a vascular complication. The mother and two sons of proband 2 also have the *COL5A1* variant, one of whom fulfils clinical criteria of cEDS. This case report highlights the significant intrafamilial variability within the cEDS phenotype. We demonstrate that patients with (likely) pathogenic *COL5A1* variants can have an absence of collagen flowers on biopsy. It is currently unclear whether the absence of collagen flowers can be linked to the (severity of) clinical features and/or the specific genetic cause. It is also uncertain whether the vascular complication in P2 is caused by the *COL5A1* variant. Absence of collagen flowers in patients with (likely) pathogenic *COL5A1* variants has not been previously reported in the literature but is important when evaluating the significance of a TEM result in patients with suspected cEDS and underscores the relevance of molecular analysis.
